# Intelligent control of mode-locked femtosecond pulses by time-stretch-assisted real-time spectral analysis

**DOI:** 10.1038/s41377-020-0251-x

**Published:** 2020-01-28

**Authors:** Guoqing Pu, Lilin Yi, Li Zhang, Chao Luo, Zhaohui Li, Weisheng Hu

**Affiliations:** 1 State Key Lab of Advanced Communication Systems and Networks, Shanghai Institute for Advanced Communication and Data Science, Shanghai Jiao Tong University, Shanghai, 200240 China; 2 Sun Yat-sen University/Southern Marine Science and Engineering Guangdong Laboratory, Zhuhai, China

**Keywords:** Imaging and sensing, Mode-locked lasers, Ultrafast photonics

## Abstract

Mode-locked fiber lasers based on nonlinear polarization evolution can generate femtosecond pulses with different pulse widths and rich spectral distributions for versatile applications through polarization tuning. However, a precise and repeatable location of a specific pulsation regime is extremely challenging. Here, by using fast spectral analysis based on a time-stretched dispersion Fourier transform as the spectral discrimination criterion, along with an intelligent polarization search algorithm, for the first time, we achieved real-time control of the spectral width and shape of mode-locked femtosecond pulses; the spectral width can be tuned from 10 to 40 nm with a resolution of ~1.47 nm, and the spectral shape can be programmed to be hyperbolic secant or triangular. Furthermore, we reveal the complex, repeatable transition dynamics of the spectrum broadening of femtosecond pulses, including five middle phases, which provides deep insight into ultrashort pulse formation that cannot be observed with traditional mode-locked lasers.

## Lasers: Fine control over very short pulses

A method providing greater control over very short duration light pulses generated by systems called mode-locked fiber lasers (MLFLs) will enhance the use of the light in such diverse applications as atomic clocks, radars, optical computing, measuring systems and astronomy. Researchers in China led by Lilin Yi at Shanghai Jiao Tong University developed apparatus and software algorithms allowing automatic ‘intelligent control’ over the generation of MLFL light pulses. The system can manipulate key aspects of the frequency range and composition of the pulses – technically their ‘spectral width’ and ‘spectral shape’ – more effectively than previously possible. The procedure also yields new technical insights into the factors determining the nature of the generated light pulses. The researchers believe their low-cost and portable system will find widespread application in research and industry.

## Introduction

Because pulse trains achieve excellent performance with a simple laser setup^1^, passively mode-locked fiber lasers (MLFLs) based on nonlinear polarization evolution (NPE) have numerous applications, ranging from high-resolution atomic clocks^2,3^ to optical frequency measurements^4,5^, photonic analog-to-digital converters (ADCs)^6^, photonic radars^7^, fine ranging metrology^8,9^, and astronomy^10,11^. However, NPE-based MLFLs are difficult to operate in the desired pulsation regime via manual polarization tuning and are prone to detaching from the desired regime due to polarization drift from environmental disturbances^12^.

To address these challenges, automatic^13–17^ or intelligent^18^ mode-locking techniques using adaptive algorithms and electric polarization controllers (EPCs) have emerged in recent years. Several automatic mode-locking lasers use temporal information to help identify the mode-locking regimes^14,17,18^. Combined with automatic optimization algorithms, such lasers can successfully reach the mode-locking regimes, but their pulse width and spectral shape are unpredictable. Thus, automatic mode-locking techniques based on a temporal discrimination alone cannot achieve mode-locking with the possible shortest pulse width and desired spectral distribution. Even though optical spectral information can be utilized in automatic mode-locking using an optical spectrum analyzer (OSA)^14–16^, such bulky and slow equipment only obtains integrated spectral information and therefore cannot be used for real-time mode-locking. A time-stretch dispersion Fourier transform (TS-DFT) produces a map between the spectrum and the temporal pulse by using a dispersive medium^19^. Combined with a real-time oscilloscope, a TS-DFT operates as a real-time OSA, resolving the issue of the low frame rate in a traditional OSA^20^. In numerous works, TS-DFT-based fast spectral analysis has been used with mode-locked lasers to observe soliton dynamics, including soliton explosions^21,22^, build-up processes of various pulsation regimes^23–25^, dissipative solitons^26^, soliton molecules^27–29^, sophisticated soliton dynamics^30–34^, and the transition dynamics between pulsation regimes^35,36^. In this article, for the first time, we propose using TS-DFT-based fast spectral analysis as the discrimination criterion to achieve rich mode-locking regimes. By simply inserting a dispersion medium into the real-time feedback loop of an automatic mode-locking laser and combining this method with an intelligent polarization search using a genetic algorithm (GA), we can manipulate the spectral width and shape of the mode-locked femtosecond pulses in real time. The spectral width of the mode-locked femtosecond pulses can be tuned from 10 to 40 nm with a resolution of ~1.47 nm, and the spectral shape can be programmed to be hyperbolic secant or triangular. With real-time control of the spectral width and shape of the mode-locking pulses, we reveal the complex and repeatable transition dynamics from the narrow-spectrum mode-locking regime to the wide-spectrum mode-locking regime, including five middle phases: a relaxation oscillation (RO), single soliton state, multi-soliton state, triangle-spectrum transition, and chaotic transition, providing deep insight into the ultrashort pulse formation that cannot be observed with traditional mode-locked lasers. We believe our proposed low-cost, portable, spectrum-programmable intelligent mode-locking fiber laser will find widespread applications in both research and industry.

## Results

### Principles of the time-stretch-assisted real-time pulse controller (TSRPC)

**Fig. 1:**
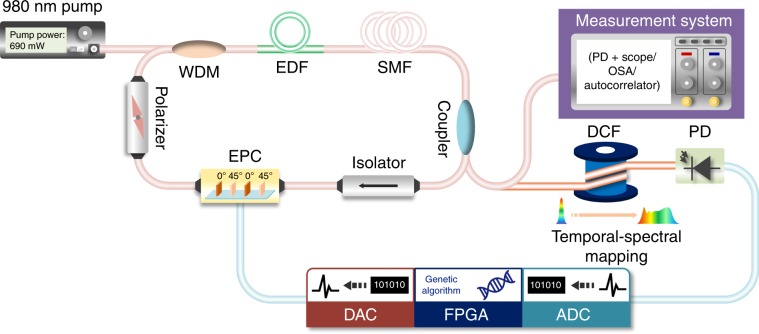
Experimental setup. The intelligent mode-locking fiber laser with an embedded time-stretch-assisted real-time pulse controller (TSRPC). The electric polarization controller (EPC) and the polarizer together produce artificially saturated absorption in nonlinear polarization evolution (NPE)-based mode locking. Part of the output power is sent to the measurement system for characterization, and the rest is used for feedback. The TSRPC consists of a dispersion compensation fiber (DCF) to complete the temporal-spectral mapping, a photodiode (PD), and a genetic algorithm-based intelligent real-time optimizer.

Figure 1 shows the experimental setup of the intelligent mode-locking fiber laser with an embedded (TSRPC. The laser cavity length is ~54.7 m, corresponding to a fundamental repetition rate of ~3.78 MHz. An 8-m erbium-doped fiber (EDF) with a dispersion of −38 ps (nm km)^−1^, serving as the gain medium, was pumped by a 980-nm laser through a wavelength division multiplexer (WDM). Considering the trade-off between the precision of the spectral control and the low sampling rate of the ADC, we inserted a ~30-m single-mode fiber (SMF) after the EDF to reduce the fundamental repetition rate. An optical coupler retains most of the power inside the cavity for the oscillation, and the rest of the power is sent out for the feedback and measurement. The isolator ensures that the laser cavity operates only in the clockwise direction. The EPC, driven by four DC voltage channels, can generate all possible polarization states over the Poincaré sphere, where each set of voltages corresponds to a specific polarization state and can respond on a timescale of microseconds (see the “Materials and methods” section). The polarizer is the core component combining the EPC to produce artificial saturable absorption in NPE-based mode-locking. The laser is a soliton laser^37^ with a net cavity dispersion of ~8.96 ps (nm km)^−1^. Part of the output power is sent to the measurement system (see the “Materials and methods” section) for characterization, and the rest is used for feedback. The lower part of Fig. 1 shows the TSRPC for the MLFLs, including a dispersion compensation fiber (DCF), a photodiode (PD), and a GA-based intelligent real-time controller (see the “Materials and methods” section). The DCF with a dispersion parameter of −1696 ps nm^−1^ in the C band is used to complete the temporal-to-spectral mapping. Thus, the theoretical precision of the spectral control, determined by both the sampling interval of the ADC and the dispersion of the DCF, is ~1.47 nm.

The core of the TSRPC is the intelligent real-time optimizer based on the GA, a global optimization algorithm inspired by species reproduction^38^. First, the GA randomly generates the initial generation. Each individual in the generation has four genes, which correspond to the four control voltages of the EPC. Then, the fitness of each individual is calculated by extracting pulses from the dispersed consecutive waveform through the slope. The fitness functions change based on the spectral shaping target. For example, the fitness function for spectral full width at half maximum (FWHM) programming is the spectral width of the current pulse, while the function for spectral shape programming is the normalized mean-squared error (NMSE) between the current pulse and the desired pulse. This method is different from traditional GA-based automatic mode-locking^13–16^, where the algorithm iterates through all the given iterations and then artificially checks if mode-locking is achieved via off-line measurements. Here, we add spectral discrimination (see the “Materials and methods” section) based on fast spectral analysis into the algorithm to judge whether the current spectrum reaches the desired target. The algorithm exits immediately when the desired spectrum is achieved, greatly reducing the algorithm run time by avoiding the remaining calculations (see the “Materials and methods” section). Then, standard genetic operations—including roulette selection, cross, mutation, and reinsertion—are performed to breed the next generation. The GA strives to understand the mapping between the polarization and fitness by iterating and then searches for the polarization with the best fitness.

### Spectral FWHM programming

**Fig. 2:**
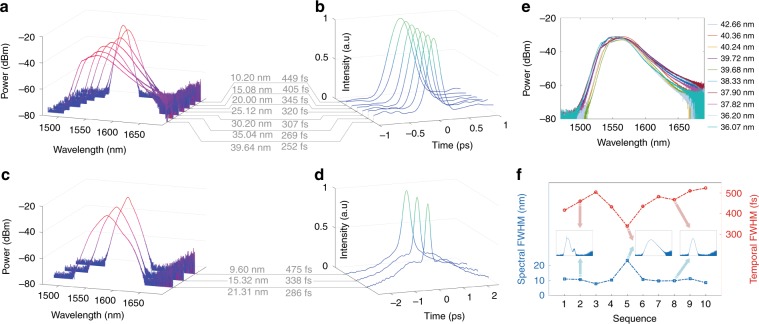
Spectral FWHM programming. **a**, **b** Spectral full width at half maximum (FWHM) programming from 10 to 40 nm with intervals of 5 nm including the spectra **a** and autocorrelation traces **b** for a fundamental repetition rate of ~3.78 MHz. **c**, **d** Spectral FWHM programming from 10 to 20 nm with equal intervals of 5 nm, including the spectra **c** and autocorrelation traces **d** for a fundamental repetition rate of ~8.6 MHz. **e** Repeatability test of searching for the maximum spectral FWHM. **f** The mode-locking results without the TSRPC.

With the TSRPC, the output spectral FWHM can be programmed. A certain FWHM range is set according to the target FWHM to precisely tune the spectral FWHM. For example, if the target spectral FWHM is 15 nm, the pre-set range is 14–16 nm. The spectrum with the target FWHM is found when the fitness of an individual falls into the pre-set range. Figure 2 shows the experimental results of the spectral FWHM programming, assessing a series of target FWHMs from 10 to 40 nm with an interval of 5 nm. The programming results are shown in Fig. 2a. The seven spectral FWHMs shown in Fig. 2a are quite near the pre-set target FWHM, indicating the validity of the TSRPC in programming the spectral FWHM. Figure 2b shows the corresponding autocorrelation trace of these spectra. This result agrees with the theory that the temporal duration decreases as the spectral width increases. Thus, the temporal duration of ultrashort pulses can also be programmed to a certain degree by using the TSRPC. Note that the measured shortest pulse width is only 252 fs because we have not optimized the cavity dispersion and because an optical amplifier is used before the autocorrelator, which may broaden the pulse width. To test the generalization of the TSRPC, we remove the ~30-m SMF inserted after the EDF in the laser cavity, increasing the fundamental repetition rate to ~8.6 MHz. Now, the laser operates as a stretched-pulse laser since the net cavity dispersion reduces to approximately −1.26 ps (nm km)^−1^, which is a near-zero dispersion value^37^. Although the maximum spectral width is reduced by half to ~20 nm, the experimental results in Fig. 2c, d reveal a concordant conclusion, further validating the TSRPC in programming the spectral width. Intriguingly, as the spectrum widens, the probability of the appearance of continuous-wave components decreases. Figure 2e shows multiple results of searching for the maximum spectral width at a fundamental repetition rate of 3.78 MHz. While searching for the maximum spectral width, there is no upper limit, and the lower limit is constantly adjusted in numerous trials until an appropriate lower limit for the maximum spectral width is found. The spectra in Fig. 2e show the same longer tail on the right side, even though the spectral width varies from 36.07 to 42.66 nm. This result demonstrates that this system is repeatable to some extent. In contrast, using our previous intelligent mode-locking method^18^ without the TSRPC, we conduct 10 consecutive measurements of the spectral FWHM and temporal FWHM of the final fundamental mode-locking regime, and the results are shown in Fig. 2f. The output spectral widths and the temporal durations are uncontrollable, as shown in Fig. 2f. The spectral widths are distributed around 10 nm, because a spectrum with an FWHM of ~10 nm is the most common fundamental mode-locking regime that corresponds to a large polarization solution space in this laser. In addition, the spectral shapes are not controllable, as shown in the three insets of Fig. 2f, and the possibility of the output spectra exhibiting strong continuous-wave components is larger than that for the TSRPC method according to experimental experience. For instance, the strong continuous-wave components in the spectrum shown in the left inset of Fig. 2f signify a non-ideal mode-locking regime.

### Spectral shape programming

The TSRPC can also be used to fit various spectral shapes. First, a dispersed standard pulse carrying spectral information is required, and different objective spectral shapes have different standard pulses. A set of experimental data (i.e., the dispersed temporal waveform) corresponding to the objective spectral shape is required to acquire the standard pulse. Notably, the fitness function in shape programming becomes the NMSE between the current pulse and the standard pulse. Then, a threshold is set as the upper limit of the NMSE. The objective spectral shape is successfully fitted when the NMSE between the current pulse and the standard pulse is lower than a pre-set threshold.

**Fig. 3:**
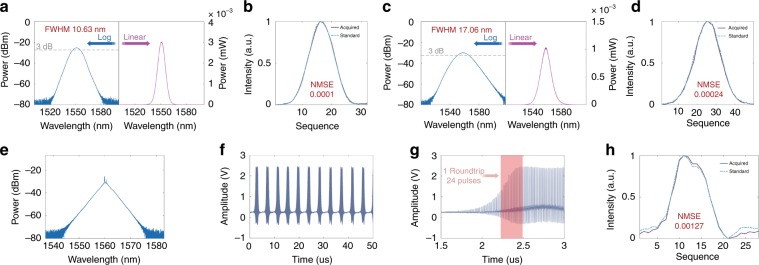
Spectral shape programming. **a**, **b** Logarithmic and linear curves of the fitted hyperbolic secant spectrum with an FWHM of 10.63 nm, with a normalized mean-squared error (NMSE) between the acquired pulse and the standard pulse of 0.0001. **c**, **d** Logarithmic and linear curves of the fitted hyperbolic secant spectrum with an FWHM of 17.06 nm, with an NMSE between the acquired pulse and the standard pulse of 0.00024. **e–h** Triangular-spectrum programming results. **e** Triangular spectrum. **f**, **g** The undispersed waveform and a magnified view. **h** The NMSE between the acquired pulse and the standard pulse is 0.00127.

Figure 3 shows the shape programming results for a fundamental repetition rate of 3.78 MHz. The upper part shows hyperbolic secant spectra programming with various standard deviations. Figure 3a shows the logarithmic and linear curves of the fitted hyperbolic secant spectrum with an FWHM of 10.63 nm. Figure 3b compares the acquired pulse after fitting with the standard pulse, showing an NMSE of only 0.0001, a nearly perfect fit. Likewise, Fig. 3c shows the logarithmic and linear curves of the fitted hyperbolic secant spectrum, with a larger FWHM of 17.06 nm. Figure 3d shows an NMSE of just 0.00024, which is a rather good fit. Notably, the spectral FWHM of the fitted hyperbolic secant spectrum fluctuates within 2 nm over multiple runs. According to experimental experience, an NMSE lower than ~0.01 usually represents a good fit. For various spectral shapes, the NMSE equivalently reveals the disparities between the current pulse and the standard pulse. Additionally, a triangular spectrum appears for the laser cavity. After extracting the standard pulse from the dispersed waveform, the triangular spectrum is reproduced by fitting the shape using the TSRPC, as shown in Fig. 3e. Figure 3f shows the undispersed waveform, and Fig. 3g shows a magnified view of the undispersed waveform. The triangular-spectrum regime corresponds to Q-switched mode-locking (QML) because its temporal waveform corresponds to the harmonic mode-locking regime modulated by low-frequency components. Thus, the triangular-spectrum regime is analogous to the breathing behavior in dissipative soliton dynamics^31^. However, the undispersed temporal envelope of the triangular-spectrum regime is trapezoidal rather than sinusoidal, as in the ordinary QML regime. There are 24 pulses within a single roundtrip of ~264.6 ns. The number of pulses within a single roundtrip is uncertain and can vary from 3 to 24 during the experiment, even when using the same standard pulse of the triangular spectrum. A comparison between the acquired pulse after fitting and the standard pulse during triangular-spectrum programming is shown in Fig. 3h. An NMSE of 0.00127 signifies a good fit. Theoretically, the output spectrum can be programmed using the TSRPC to be any spectral shape (including soliton bound states^27–29^) existing in the laser cavity but only if an ADC with a higher sampling rate or larger dispersion is used to improve the spectral resolution. Furthermore, combined with a coherent time–stretch transformation^39–42^, the phase information could be acquired, thus providing the possibility to control the phase of the pulses.

### Transition dynamics of the spectral width optimization process

Through the TS-DFT, more dynamics in MLFLs have come to light in recent studies^21–35^, deepening the understanding of the internal dynamics of mode-locking pulse formation and assisting the design of MLFLs. The starting dynamics, from the initial noise fluctuations to various regimes, have been intensively reported^23–29^, but the regime transition dynamics, which are essential processes in mode-locked lasers, are poorly understood. The transition dynamics from Q-switching to mode-locking in a soliton laser have been revealed^34^, and the transition dynamics of the Q-switching, fundamental mode-locking, and harmonic mode-locking regimes^35^ have also been observed recently. For NPE-based MLFLs, their mode-locking regimes fall into a large polarization space of different spectral widths and shapes. The evolution from the narrow-spectrum mode-locking regime to the wide-spectrum mode-locking regime is an essential physical process in producing ultrashort femtosecond pulses, but the corresponding transition dynamics have never been revealed because the physical process is very difficult to control and track in traditional mode-locking lasers. By using the TSRPC to control the spectral width and shape of the mode-locked pulses in real time, however, we revealed the transition dynamics from the narrow-spectrum mode-locking regime to the wide-spectrum mode-locking regime and from the triangular-spectrum QML regime (see Supplementary Information) to the wide-spectrum mode-locking regime by recording several sets of experienced voltages of the EPC that led to the different regimes. All of the transitions were produced at a fundamental repetition rate of 3.78 MHz, signifying a roundtrip of ~264.6 ns.

**Fig. 4:**
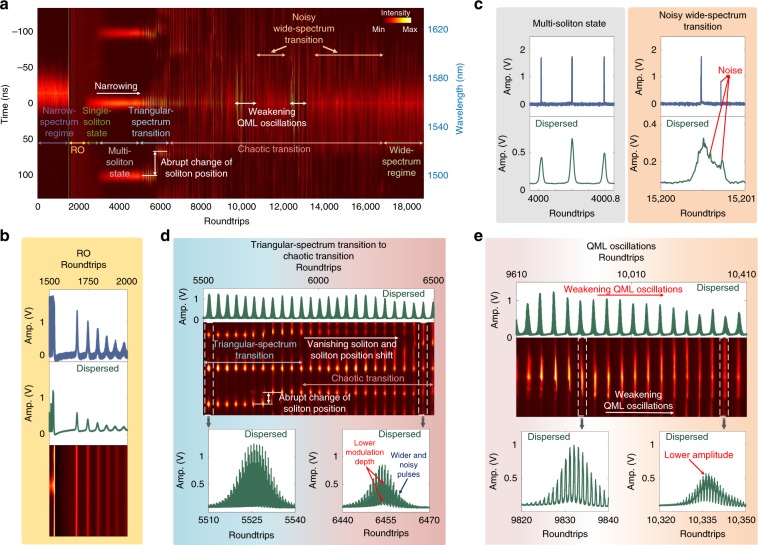
The transition dynamics from the narrow-spectrum mode-locking regime to the wide-spectrum mode-locking regime. **a** The entire transition from the narrow-spectrum regime to the wide-spectrum regime, showing complex dynamics. **b** The relaxation oscillation (RO) state induced by the polarization assignment of the EPC. **c** The multi-soliton state with three solitons in a single roundtrip and the noisy wide-spectrum transition at the 15201th roundtrip, where a noisy pulse appears on the right, adding noise to the short-wavelength range of the real-time spectrum. **d** The dynamics from the triangular-spectrum transition to the chaotic transition where an abrupt change in the soliton position, a vanishing soliton, and a soliton position shift appear. Comparing one stripe from the triangular-spectrum regime and one stripe that resembles a mixture of the triangular-spectrum transition and the Q-switched mode-locking (QML) oscillations from the chaotic transition, the latter stripe has a lower modulation depth and comprises wider and noisy pulses, blurring the sparkles. **e** QML oscillations weaken during the conversion to the noisy wide-spectrum transition due to power reallocation.

We investigated the transition dynamics from the narrow-spectrum mode-locking regime to the wide-spectrum mode-locking regime, as shown in Fig. 4. The initial spectral FWHM of the narrow-spectrum mode-locking regime and the ultimate spectral FWHM of the wide-spectrum mode-locking regime were 15.76 and 31.04 nm, respectively. The entire transition contains five middle phases, which are sequentially the RO, single-soliton state, multi-soliton state, transient triangular-spectrum transition, and chaotic transition including the QML oscillations and the unstable noisy wide-spectrum transition, as shown in Fig. 4a. Figure 4b shows the RO induced by the polarization assignment of the EPC. After the RO dies out, the laser enters a short-lived single-soliton state with a noisy background caused by the residual RO. Then, two solitons grow on the noisy background, forming the multi-soliton state. The solitons are generated through the RO, as previously demonstrated^23,29^. The multi-soliton state has three nearly isometric solitons in a single roundtrip, as shown on the left side of Fig. 4c. It is obvious that the middle soliton has the maximum power. One possible reason for this result is that the soliton with the maximum power corresponds to a spectral range that is inside the spectral range of the narrow-spectrum regime. Therefore, more power is preserved, forming the strongest soliton. Another reason might be that the spectral range of the strongest soliton is near the center wavelength of the ultimate wide-spectrum regime. More power is required for wavelengths inside the spectral range of the ultimate regime to complete the transition. The situation in Fig. S1 also supports these two reasons (see Supplementary Information). However, for the multi-soliton state, the temporal amplitude constantly increases due to spectral narrowing of some solitons, such as the middle soliton of the multi-soliton state in Fig. 4a. Thus, the multi-soliton state becomes unstable due to the stronger temporal amplitude and environmental disturbances.

After maintaining the multi-soliton state for ~2000 roundtrips, the temporal amplitude finally starts fluctuating, and the laser evolves into the triangular-spectrum transition of three pulses in a single roundtrip. The instability originating from the increasing temporal amplitude does not cease and pushes the laser further into the chaotic transition, as shown in Fig. 4d. The soliton changes position abruptly around the 5900th roundtrip inside the triangular-spectrum transition (see Supplementary Information), revealing the complexity of the transition dynamics. Afterwards, both the upper soliton and lower soliton vanish, and the upper soliton simultaneously changes position. Soliton decay and position shift have also appeared in transient dissipative soliton dynamics^31^.

For a comparison, two stripes selected from the triangular-spectrum transition and the chaotic transition are shown at the bottom of Fig. 4d. The latter stripe, with three blurred sparkles, looks like a mixture of the triangular-spectrum transition and the QML oscillations, as shown in Fig. 4e. Different from the RO, the interferogram of Fig. 4e shows an obvious sparkle in each envelope due to the pulses on the envelope. The QML oscillations are also unlike the triangular-spectrum regime because there is only one pulse in a single roundtrip in the QML oscillations. Comparing the two dispersed waveforms, the latter stripe has a lower modulation depth and comprises wider and noisy pulses, blurring the sparkles. The mixture of the triangular-spectrum transition and the QML oscillations holds for nearly 3000 roundtrips in the chaotic transition until it completely turns into QML oscillations. The QML oscillations, which reallocate power, evidently weaken during the conversion to the noisy wide-spectrum transition, as shown in Fig. 4e. The result of the power reallocation—the noisy wide-spectrum transition, in this case—is primarily determined by the status of the laser cavity, that is, the polarization. Before entering the noisy wide-spectrum transition, the weakened QML oscillations have a lower amplitude and far noisier pulses than the stronger QML oscillations induced by the power reallocation, as shown at the bottom of Fig. 4e. However, due to internal instabilities (i.e., nonlinear effects) and environmental disturbances, the power reallocation—the QML oscillations—can repeatedly emerge until the spectrum ultimately settles down, as shown in Fig. 4a. Through two rounds of power reallocation, the laser sequentially enters the noisy wide-spectrum transition and remains there until the ultimate wide-spectrum regime dominates. The noisy wide-spectrum transition, shown on the right side of Fig. 4c, has a noise pulse on the right side, providing considerable noise in the short-wavelength range of the real-time spectrum.

Furthermore, the transition from the triangular-spectrum regime to the wide-spectrum mode-locking regime, determined by the same set of EPC controlling voltages (i.e., the terminal polarization), shows dynamics analogous to the transition in Fig. 4 (see Supplementary Information). In conclusion, the transition dynamics depend considerably on the concrete objective regime, which is controlled by the terminal polarization in the experiments. We conducted multiple observations with the same original and terminal polarization states, and although no two transitions showed identical dynamics, all the transitions showed nearly the same middle phases, demonstrating that the transition dynamics observations were repeatable.

## Discussion

Using TS-DFT-based fast spectral analysis, we demonstrated the TSRPC method, which helps NPE-based MLFLs spectrally program the output pulse train. Benefitting from the TS-DFT and the real-time GA optimizer, the TSRPC overcomes the considerable slowness, cost, and bulkiness of traditional OSAs used in previous automatic mode-locking lasers^14–16^. The TSRPC can be made even more portable by replacing the DCF with a small optical grating, and its spectral programming resolution can be improved by using an ADC with a higher sampling rate or a medium with large dispersion. Moreover, we revealed the transitions from the narrow-spectrum mode-locking regime and from the triangular-spectrum QML regime (see Supplementary Information) to the wide-spectrum mode-locking regime with intriguing and complex dynamics. These results showed that the transition dynamics are mostly determined by the terminal polarization, regardless of the original polarization. The TSRPC, which can program the spectral FWHM and spectral shape, greatly broadens the applications of NPE-based MLFLs and undoubtedly contributes to NPE-based MLFLs.

## Materials and methods

### The EPC

Inside the EPC, there are four phase retarders, and their optical axes are oriented at 0° and 45°. Each phase retarder is individually controlled through a DC voltage and can induce a phase delay of 0 to π radians almost continuously. Hence, each phase retarder functions as a variable waveplate. The light is coupled into and out of the EPC through two collimators.

### Experimental setup

An ADC, a field-programmable gate array (FPGA), and four digital-to-analog converters (DACs) controlling the four channels of the EPC constitute the GA-based intelligent real-time controller. The 400-MSa s^−1^ ADC in the GA-based real-time optimizer has an 8-bit resolution and a maximum input amplitude of 1.2 V_pp_. Four 100-MSa s^−1^ DACs with 12-bit resolution control the four channels of the EPC. Thus, the size of the polarization space reaches 4096^4^. The instrument used to characterize the temporal and spectral features has three parts: to characterize the temporal features, a 10-GHz PD and a real-time oscilloscope with a sampling rate of 2.5 GSa s^−1^ capture the temporal waveforms. The temporal duration of the output pulses is measured by an auto-correlator, and the spectra of the output pulses are measured using a traditional OSA.

A high-speed real-time oscilloscope with a sampling rate of 10 GSa s^−1^ measures both the undispersed and dispersed data in the transition observations, producing a spectral resolution of ~0.06 nm. Additionally, the data acquisition of the transition observations is ensured by the trigger scheme achieved via the FPGA. When the EPC is assigned a set of new voltages, the FPGA will send out a trigger signal to inform the oscilloscope that the transition has begun.

### Spectral discrimination in the GA

For both spectral FWHM programming and spectral shape programming, the fitness of each individual (i.e., each polarization or each set of DAC output voltages) inside the GA is not determined by the fitness of a single stretched pulse (i.e., a single real-time spectrum) but is determined by the mean fitness of several stretched pulses that are sampled at different times. The aim behind this choice is to improve the accuracy when assessing each polarization. When the mean fitness surpasses the pre-set threshold for the first time, the spectrum has not yet been considered to be one of the target spectra. The algorithm is then delayed for several seconds. Then, a new pulse train is sampled by the ADC, and a second check is performed to ensure that most of the various transient instabilities^43^ can be filtered. When the fitness calculated from the new pulse train passes the second check (i.e., surpassing the pre-set threshold), the spectrum is finally considered to be one of the target spectra, and the current polarization is considered to be one solution. After successfully searching one solution, the algorithm terminates, thereby skipping the calculation of the remaining generations in the middle of the GA optimization to reduce the algorithm running time.

## Supplementary information

Supplementary Information for “Intelligent control of mode-locked femtosecond pulses by time-stretch-assisted real-time spectral analysis”
