# Supplementary material for: Intelligent control of mode-locked femtosecond pulses by time-stretch-assisted real-time spectral analysis
Source: Light Sci Appl. 2020 Jan 28;9:13. doi: 10.1038/s41377-020-0251-x (PMC6987192; doi:10.1038/s41377-020-0251-x)
Supplement: Supplementary file 1 — Supplementary Information for “Intelligent control of mode-locked femtosecond pulses by time-stretch-assisted real-time spectral analysis” [file 41377_2020_251_MOESM1_ESM.docx]

**Supplementary Information for “Intelligent control of mode-locked femtosecond pulses by time-stretch-assisted real-time spectral analysis”**

Guoqing Pu^1^, Lilin Yi^1^*, Li Zhang^1^, Chao Luo^1^, Zhaohui Li^2^ and Weisheng Hu^1^

^1^State Key Lab of Advanced Communication Systems and Networks, Shanghai Institute for Advanced Communication and Data Science, Shanghai Jiao Tong University, Shanghai, 200240, China

^2^Sun Yat-sen University/Southern Marine Science and Engineering Guangdong Laboratory (Zhuhai), China

*Corresponding author: lilinyi@sjtu.edu.cn

Fig. S1 shows the transition dynamics from the triangular-spectrum regime to the wide-spectrum mode-locking regime. The wide-spectrum mode-locking regime is determined by the same set of EPC controlling voltages (i.e., the terminal polarization), and the middle phases of this transition quite resemble the transition shown in Fig. 4a. The triangular-spectrum regime has four pulses in a single roundtrip because there are four longitudinally equidistant sparkles in a single roundtrip, as shown in the interferogram of Fig. S1b. This figure also shows the temporal waveform with a trapezoidal envelope and its dispersed version. The dispersed temporal waveform is quite analogous to the usual QML regime with a sinusoidal envelope and, as expected, corresponds to the constellation-like interferogram where the horizontal margin between two sparkles is the envelope period of the temporal waveforms. Fig. S1c shows the RO induced by the polarization assignment of the EPC. Similarly, solitons generated via the RO^1,2^ form the sequential single-soliton state and multi-soliton state. Fig. S1d shows one roundtrip of the multi-soliton state where the positions of the three solitons are rather certain and stable. Clearly, the lower soliton has the maximum power. Two possible reasons behind this have already been introduced in the main content. First, the spectral range of the strongest soliton is near the spectral range of the second pulse in the triangular-spectrum regime. The spectral ranges of the upper two solitons near the spectral range of the first pulse in the triangular-spectrum regime. However, the power is split since two solitons grow up simultaneously. Second, the spectral range of the strongest soliton is completely covered by the spectral range of the ultimate wide-spectrum regime. Then, the spectra narrowing also appears in the multi-soliton state, so the temporal amplitude constantly increases. Because of the stronger temporal amplitude and environmental disturbances, the multi-soliton state changes into the chaotic transition through a short-lived triangular-spectrum transition of three pulses in a single roundtrip. The QML oscillations in the chaotic transition shown in Fig. S1e cause power reallocation. Fig. S1f shows the transition dynamics from a triangular spectrum to a wide spectrum. Sequentially, the middle soliton vanishes and the rest of the solitons shift in position over time, and these phenomena also appear in the transient dissipative soliton dynamics^3^. Until the characteristics of the triangular-spectrum transition completely disappear, the QML oscillation reallocates the power inside the cavity and the laser sequentially enters the wide-spectrum transition. The whole transition completes around the 5600th roundtrip, right after the chaotic transition. Overall, the dynamics in this transition are rather similar to the transition dynamics shown in Fig. 4.


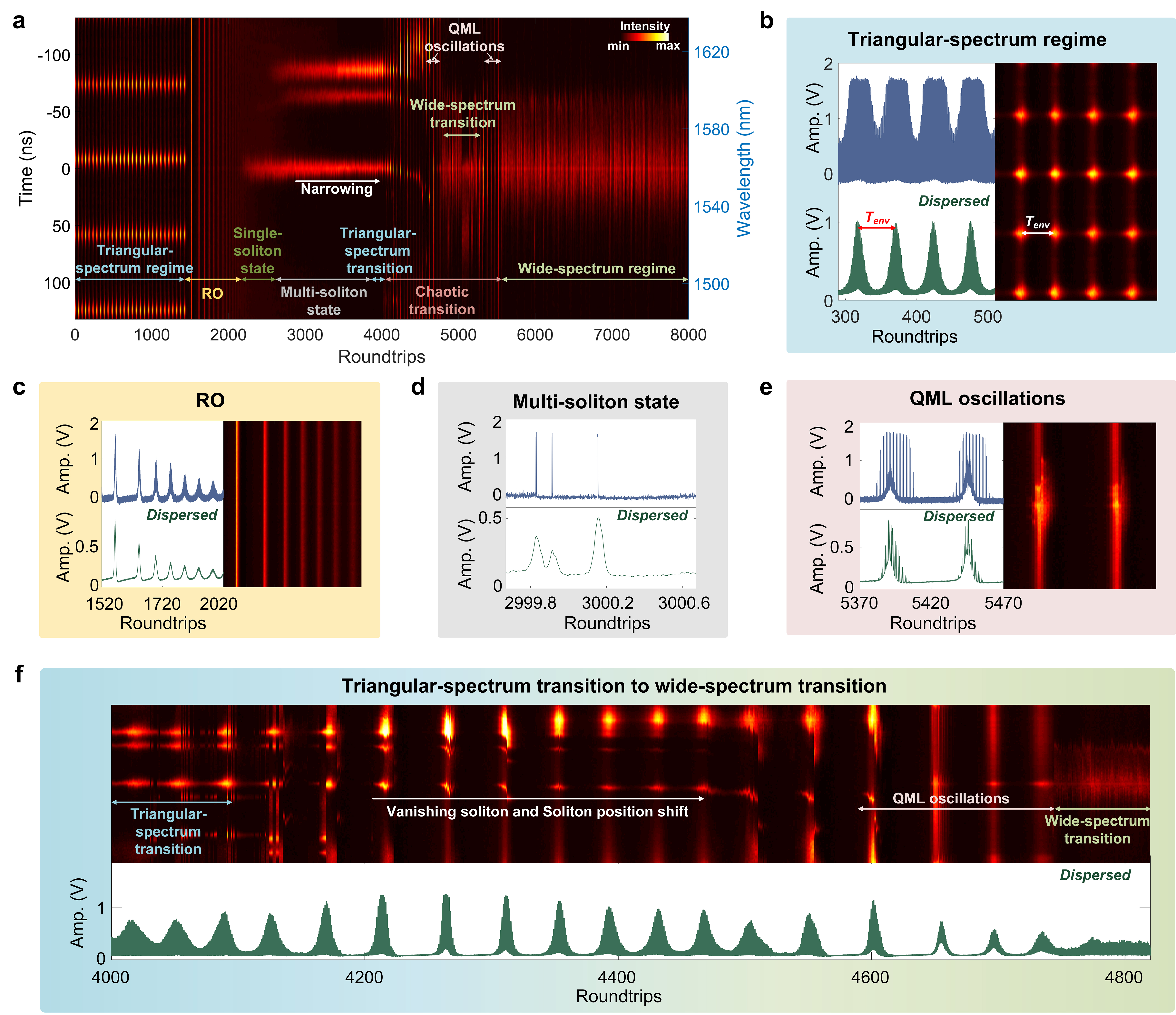


**Fig. S1 | The transition from the triangular-spectrum regime to the wide-spectrum mode-locking regime with complex dynamics. a**, The entire transition, showing the complex dynamics. **b**, The triangular spectrum with the temporal waveform at the top-left, the dispersed waveform at the bottom-left, and the corresponding interferogram on the right. **c**, The RO induced by the polarization assignment of the EPC. **d**, The multi-soliton state with three solitons in a single roundtrip. **e**, The QML oscillations inside the chaotic transition. **f**, The transition dynamics from a triangular spectrum to a wide spectrum, showing a vanishing soliton and soliton position shift.

References

1. Herink, G. *et al*. Resolving the build-up of femtosecond mode-locking with single-shot spectroscopy at 90 MHz frame rate. *Nature Photonics* **10**, 321-326 (2016).
2. Peng, J. S., & Zeng, H. P. Build‐Up of dissipative optical soliton molecules via diverse soliton interactions. *Laser & Photonics Reviews* **12**, 1800009 (2018).
3. Ryczkowski, P. *et al*. Real-time full-field characterization of transient dissipative soliton dynamics in a mode-locked laser. *Nature Photonics* **12**, 221-227 (2018).
